# Bronchoalveolar Lavage Fluid from Chronic Obstructive Pulmonary Disease Patients Increases Neutrophil Chemotaxis Measured by a Microfluidic Platform

**DOI:** 10.3390/mi14091740

**Published:** 2023-09-06

**Authors:** Jiaqi Ren, Wenfang Chen, Zhicheng Zhong, Ning Wang, Xi Chen, Hui Yang, Jing Li, Ping Tang, Yanping Fan, Francis Lin, Changqing Bai, Jiandong Wu

**Affiliations:** 1 Institute of Biomedical and Health Engineering, Shenzhen Institute of Advanced Technology, Chinese Academy of Sciences, Shenzhen 518055, China; 2 Department of Pulmonary and Critical Care Medicine, Shenzhen University General Hospital, Shenzhen 518055, China; 3 School of Optical-Electrical and Computer Engineering, University of Shanghai for Science and Technology, Shanghai 200093, China; 4 Department of Physics and Astronomy, University of Manitoba, Winnipeg, MB R3T 2N2, Canada

**Keywords:** microfluidics, cell migration, neutrophil chemotaxis, COPD, bronchoalveolar lavage fluid

## Abstract

Chronic obstructive pulmonary disease (COPD) is a persistent and progressive respiratory disorder characterized by expiratory airflow limitation caused by chronic inflammation. Evidence has shown that COPD is correlated with neutrophil chemotaxis towards the airways, resulting in neutrophilic airway inflammation. This study aimed to evaluate neutrophil chemotaxis in bronchoalveolar lavage fluid (BALF) from COPD patients using a high-throughput nine-unit microfluidic platform and explore the possible correlations between neutrophil migratory dynamics and COPD development. The results showed that BALF from COPD patients induced stronger neutrophil chemotaxis than the Control BALF. Our results also showed that the chemotactic migration of neutrophils isolated from the blood of COPD patients was not significantly different from neutrophils from healthy controls, and neutrophil migration in three known chemoattractants (fMLP, IL-8, and LTB4) was not affected by glucocorticoid treatment. Moreover, comparison with clinical data showed a trend of a negative relationship between neutrophil migration chemotactic index (C. I.) in COPD BALF and patient’s spirometry data, suggesting a potential correlation between neutrophil migration and the severity of COPD. The present study demonstrated the feasibility of using the microfluidic platform to assess neutrophil chemotaxis in COPD pathogenesis, and it may serve as a potential marker for COPD evaluation in the future.

## 1. Introduction

Chronic obstructive pulmonary disease (COPD) is the third leading cause of morbidity and mortality worldwide, according to the World Health Organization, causing 3.23 million deaths in 2019 [1]. It is a persistent and progressive respiratory disorder characterized by expiratory airflow limitation caused by chronic inflammation in peripheral airways and lung parenchyma and excessive mucus and chemokine secretion, which reduce lung function and lead to hypoxic respiratory failure [2,3,4,5,6]. Patients with COPD usually suffer from breathing difficulties, a persistent cough, and phlegm production. Long-term exposure to harmful gases, particles, dusts, fumes, chemicals, and tobacco smoke are the main etiological factors in the pathogenesis of COPD [7,8,9]. At the cellular level, this chronic inflammation is characterized by an increased number of inflammatory cells, including alveolar macrophages, T lymphocytes, innate lymphoid cells, and neutrophils traveling along the circulation [10]. They secrete multiple inflammatory mediators that attract more inflammatory cells from the circulation, thus amplifying the inflammatory response and inducing structural changes [11].

Neutrophils are the most abundant leukocyte in human circulation, accounting for approximately 50–70% of total white blood cells. They are crucial for the innate immune response and are capable of modulating the adaptive immune response [12]. “End-target” chemoattractants such as N-formyl-methionyl-leucyl-phenylalanine (fMLP) and “intermediate” chemoattractants such as interleukin 8 (IL-8) and leukotriene B4 (LTB4) trigger hierarchical chemotactic signals that attract neutrophils to the inflammatory sites [13,14]. In terms of pulmonary inflammation, neutrophils have been shown to promote the clearance of nuclear debris following tissue injury [15]. The dysregulation of neutrophils contributes to various chronic lung diseases, such as asthma, COPD, and idiopathic pulmonary fibrosis (IPF) [16]. Neutrophilic airway inflammation is a predominant feature of COPD [17]. The increased activity of neutrophils in the bronchial wall lumen of COPD patients was correlated with airway obstruction and declined lung function, including decreased forced expiratory volume in 1 s (FEV_1_) and reduced gas transfer [18,19,20,21,22]. Evidence has shown that neutrophils are the most abundant blood cell in the sputum or bronchoalveolar lavage fluid (BALF) of patients with both stable and exacerbated COPD [23,24], and the increased sputum neutrophilia is correlated to airway obstruction [22,25,26]. Therefore, the measurement of neutrophil counts or chemotactic factors recruiting neutrophils has become a potential diagnostic marker for COPD [27,28].

**Figure 1:**
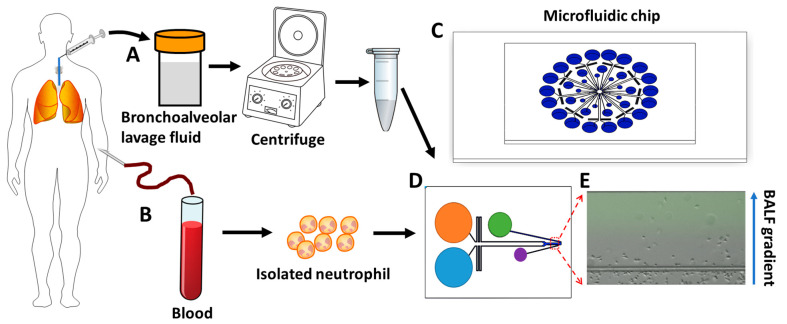
Illustration of the process of using a microfluidic device for testing neutrophil chemotaxis in the BALF gradient. (**A**) BALF collection and preparation. (**B**) Blood collection and neutrophil isolation. (**C**) The nine-unit radial microfluidic cell migration device. (**D**) The single detection unit of the nine-unit device. (**E**) A representative image of neutrophils in a COPD BALF gradient in the microfluidic channel at the end of the 30 min chemotaxis experiment. BALF: bronchoalveolar lavage fluid.

Cell migration plays an important role in the development and maintenance of multicellular organisms, and cell migration studies are well-known for assisting in the diagnosis of various diseases [29,30]. The traditional cell migration assays (e.g., transwell assay and under-agarose assay) were limited by their inability to control gradient, making it difficult to obtain reliable chemotaxis results [31,32]. The Boyden chamber, developed in 1962, was considered the gold standard technique for studying cell chemotaxis [33]; however, the concentration gradient generated is steep and unstable, and it is difficult to distinguish between cell chemotaxis and migration. Later, the Dunn chamber, developed from the Boyden chamber, was widely used to study cell chemotaxis [34]. However, the concentration gradient in the Dunn chamber is generated by flow-free diffusion, which disappears quickly, making it unable to maintain a stable concentration gradient for cell chemotaxis. In recent decades, the development of microfluidics has revolutionized research in cell migration with its low research cost, high throughput and portability, and the capability of precise manipulation of various parameters that influence cell migration [35,36,37,38,39]. The function of cell migration in chronic respiratory diseases such as asthma and COPD has been previously tested using microfluidic technology, which validated the feasibility of using this platform as a new characterization tool for these diseases [27,40]. Sackmann et al. utilized a microfluidic chip to study neutrophil migration in asthma patients and concluded that neutrophil chemotaxis velocity could be a potential biomarker for asthma [40]. Wu et al. utilized a microfluidic platform to study neutrophil chemotaxis induced by COPD sputum samples and found increased neutrophil chemotaxis in COPD sputum compared to control sputum [27]. Compared to sputum, the BALF sample is more representative of the internal lung microenvironment. Evidence suggests increased neutrophils in COPD BALF, and neutrophils from COPD patients have an elevated velocity [21,24,41]. However, the migratory behaviors of neutrophils in COPD BALF are not well characterized. In the present study, we used a radial nine-unit microfluidic platform to evaluate neutrophil chemotaxis in BALF samples from COPD patients (Figure 1) and compared the migration behaviors of neutrophils isolated from COPD patients and controls. We hypothesized that BALF samples from COPD patients would promote neutrophil migration and might become a potential clinical assessment for COPD.

## 2. Methods

### 2.1. Bronchoalveolar Lavage Fluid (BALF) Sample Collection and Preparation

Fifteen participants (8 COPD patients and 7 control subjects) were recruited from the Shenzhen University General Hospital (Table 1). The COPD patients were diagnosed based on the spirometry data and physician evaluation according to the 2022 Global Initiative for Chronic Lung Disease (GOLD) criteria. In brief, patients with clinically stable COPD with pulmonary function showing post-bronchodilator FEV1/FVC < 70% were recruited. And patients with a history of asthma, allergies, positive RAST tests for common allergens in the blood, or alpha-1 antitrypsin deficiency were excluded from the cohort. The detailed clinical information of the COPD participants can be found in Supplementary Table S1. The protocol (KYLL-20221212A) was approved by the Ethics Board at Shenzhen University General Hospital, and researchers adhered to the principles of the Helsinki Declaration over the course of the study.

Radiological imaging was performed prior to the collection of BALF to localize the focus of the disease. Then, the bronchoscope was guided to the specific region identified by the radiological imaging and wedged into the subsegment, ideally with visualization of the distal airway in the center of the image. In the case of diffuse lesions, the most preferred location for lavage would be the right middle lobe, or the lingula.

Bronchoalveolar lavage was performed under anesthesia. 20–50 mL of sterile normal saline at room temperature was injected through a hand-held syringe, then gradually drawn back into the syringe. This process was repeated 3–5 times for a total perfusion of up to 300 mL. 30% or more of the refluxed sample is considered adequate reflux, of which at least 10–20 mL is required for cellular, cytokine, and infectious analyses.

The collected BALF sample was then transferred to a sterile 15 mL centrifuge tube and centrifuged at 1000× *g* for 20 min at 4 °C to completely remove debris. The supernatant was collected and stored at −80 °C prior to the migration experiment.

### 2.2. Neutrophil Preparation

Neutrophils were collected from both healthy control blood donors and COPD blood donors. Ethics approval (KYLL-20221212A) was granted by the Ethics Board at Shenzhen University General Hospital. Neutrophils were isolated from 1 mL of blood using EasySep™ Direct Human Neutrophil Isolation Kit combined with EasySep™ Magnet (STEMCELL Technologies, Vancouver, BC, Canada) following the protocol provided by the company, which allows direct isolation of neutrophils from the whole blood within 30 min. A 2–2.5 mL sample was collected after isolation; the migration medium (RPMI-1640 with 0.4% BSA) was then added to the sample, followed by centrifugation at 300× *g* for 8 min. The supernatant was removed, and neutrophils were re-suspended in 1 mL of migration medium, resulting in a cell count of 7.25 × 10^5^ cell mL^−1^. Neutrophil viability was tested with Trypan Blue, which showed that >99% of cells were viable. Neutrophils were used for experiments within 2 h after isolation.

### 2.3. Microfluidic Device Preparation

The microfluidic device was fabricated in polydimethylsiloxane (PDMS) (Sylgard 184; Dow Corning, Midland, MI, USA) using the standard photolithography and soft-lithography procedures [37,42]. The device contained nine individual units oriented in a radial shape; each unit has a chemoattractant loading inlet, a medium loading inlet, a cell loading inlet, an outlet, and a fluid channel network (285 μm in width for the gradient channel, 100 μm in width for the branched flow channel) (Figure 1C,D). The equilibrated flow from the chemoattractant loading inlet and medium loading inlet allowed the generation of a stable gradient in the migration channel using passive pumping and pressure-balancing strategies.

The design pattern was printed on a chrome photomask at high resolution. The design was then patterned in SU-8 photoresist on a 4-inch silicon wafer by two-layer contact photolithography. The first layer was the cell docking structure, which is used to align the neutrophils to one side of the gradient channel before migration. The height of the channel was ~2.6 μm. The second layer (~55 μm) defines the gradient generation and cell migration channel. The photoresist pattern was then used as a positive mold to generate PDMS replicas using soft lithography. Specifically, PDMS and curing agent were mixed in a 10:1 ratio by weight and poured onto the master mold in a Petri dish to replicate the feature. The PDMS replica were placed in a vacuum desiccator to remove air bubbles, followed by baking at 80 °C for 1.5 h, which were then peeled off from the silicon master mold. The inlets (6 mm diameter), outlets (4 mm diameter), and cell loading port (2 mm diameter) were punched on the PDMS devices. The channel surface of the PDMS replica was cleaned with adhesive scotch tape to remove dust and PDMS residual fragments. The PDMS replica and a glass slide were then placed inside a plasma cleaner chamber for air plasma treatment for 2 min. After the treatment, the PDMS replica was bonded to the glass slide to seal the microfluidic channel. The microfluidic devices were coated using rat tail collagen type I (2 μg mL^−1^; 354236; Corning, Corning, NY, USA) for 1 h and then blocked with 0.4% BSA (Solarbio, Beijing, China) in RPMI 1640 medium for another hour at room temperature before the migration experiment.

### 2.4. Microfluidic Chemotaxis Experiment

N-Formylmethionine-leucyl-phenylalanine (fMLP; F3506; Sigma-Aldrich, Burlington, MA, USA), recombinant human Interleukin-8 (IL-8; 200-08M; PeproTech, Thermo Fisher Scientific, Waltham, MA, USA), and Leukotriene B4 (LTB4, L0517; Sigma-Aldrich, USA) diluted with the migration medium (RPMI-1640 with 0.4% BSA) to a final concentration of 100 nM were included as chemoattractants for positive controls in neutrophil migration experiments. The procedure for cell and solution addition onto the microfluidic chip was previously described [37]. Briefly, the neutrophil suspension was added to fill the cell loading port of each unit of the pre-treated device, and cell movement toward the docking area was observed under a microscope. When adequate neutrophils were collected at the docking area, ~70 µL test solution (fMLP, IL-8, LTB4, or BALF samples) and migration medium were added to the corresponding inlets. The inlet that is closer to the cell loading port was filled with migration medium, and the other inlet was filled with test solutions (chemoattractant or BALF samples). Each set of conditions was replicated at least 3 times. The inlets of each unit were then connected using silicone oil (146153; Sigma-Aldrich, USA) to balance the pressure difference [37]. FITC-dextran (10 kDa; FD-10S; Sigma-Aldrich, USA) was added to the chemoattractant solutions and BALF to characterize proper gradient generation.

The microfluidic device was then placed on a microscope stage enclosed by an environmental control chamber, which maintained the temperature at 37 °C. The humidity was also maintained by adding deionized water to the water channel surrounding the microscope stage inside the environmental chamber. The chemotactic migration of neutrophils was observed using an inverted fluorescence microscope (Nikon ECLIPSE Ti2-E, Tokyo, Japan). The time-lapse differential interference contrast (DIC) and fluorescent images of neutrophil migration for each of the 9 units were captured every 30 s for 30 min.

### 2.5. Drug Response Experiment

Dexamethasone sodium phosphate injection (100 μg mL^−1^) was used for the drug response experiment. There were two parts to the experiment. For the first part, dexamethasone was added to the prepared fMLP (100 nM), IL-8 (100 nM), and LTB4 (100 nM) solutions, and neutrophil migration was tested in the microfluidic device as previously described. For the second part of the drug response experiment, the isolated neutrophils were treated with dexamethasone (100 μg mL^−1^) for 1 h prior, and their migration in IL-8, fMLP, and LTB4 gradient fields was tested, respectively, as previously described.

### 2.6. Data Analysis

The neutrophil migratory behaviors in different experimental conditions were quantified by previously established manual tracking methods [39]. Briefly, the representative neutrophil trajectories were tracked using the Manual Tracking plugin for the ImageJ software (https://imagej.nih.gov/ij/download.html (accessed on 23 March 2022). The manually tracked results were then input into the Chemotaxis & Migration Tool version 2.0 to calculate (1) the chemotactic index (C. I.), which is the ratio of cell displacement toward the chemoattractant gradient to the total migration distance; and (2) the average migration velocity, calculated as the ratio of total migration distance to the experiment period. Data were expressed as means ± standard error of the mean (SEM). Statistical analyses were performed with an unpaired Student’s two-tailed *t*-test (for 2 groups) and a one-way ANOVA with post hoc Tukey’s multiple comparisons test (for more than 2 groups) if the data were normally distributed, and a Kruskal–Wallis test with post hoc Dunn’s multiple comparisons test if the data were not normally distributed. Linear regression analyses were performed to assess the relationship between neutrophil migration data and clinical data. *p* ≤ 0.05 (*) was considered statistically significant.

## 3. Results

### 3.1. Neutrophil Chemotaxis in COPD BALF, Control BALF, and Known Recombinant Chemoattractants

**Figure 2:**
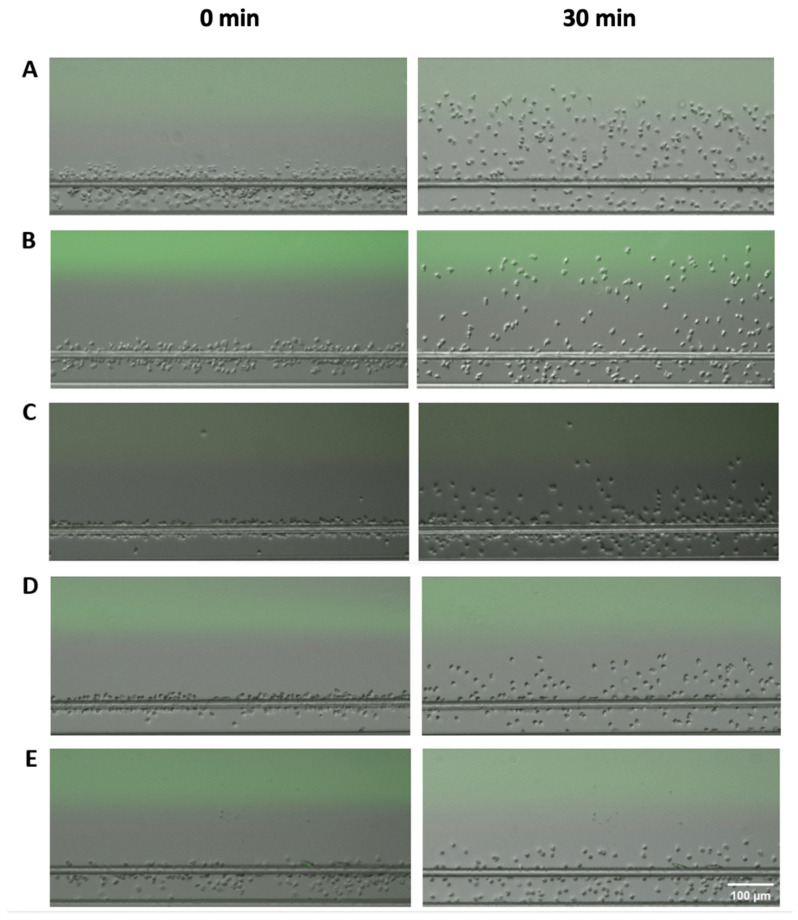
Microscopic images of neutrophil migration under different conditions at the beginning (**left**) and at the end of the experiment (**right**). (**A**) Neutrophil migration in the fMLP gradient; (**B**) neutrophil migration in the IL-8 gradient; (**C**) neutrophil migration in the LTB4 gradient; (**D**) neutrophil migration in the COPD BALF gradient; (**E**) neutrophil migration in the Control BALF gradient.

**Figure 3:**
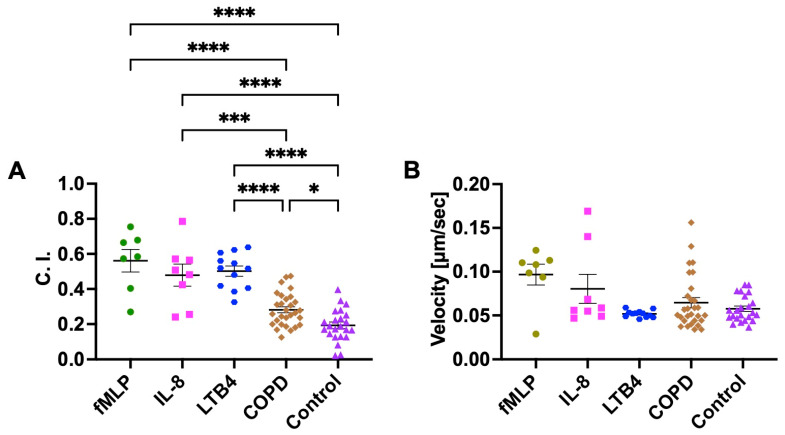
Neutrophil chemotaxis in fMLP, IL-8, LTB4, and different BALF samples (COPD or Control) in the microfluidic device. Neutrophil migration C. I. (**A**) and velocity (**B**) in different fMLP, IL-8, LTB4, and BALF sample gradient fields. Data presented as mean ± SEM. ‘*’ indicates *p* < 0.05; ‘***’ indicates *p* < 0.001; ‘****’ indicates *p* < 0.0001.

We first used neutrophils isolated from the blood of healthy controls and evaluated their migratory dynamics in different chemical gradients, including the known neutrophil chemoattractants (fMLP, IL-8, and LTB4), COPD BALF, and Control BALF (Figure 2). As expected, the neutrophils showed strong chemotaxis in gradients of all three known chemoattractants. The C. I. was significantly higher in fMLP, IL-8, and LTB4 gradients compared to COPD and Control BALF gradients (Figure 3A). We then compared the migratory dynamics of neutrophils in different BALF samples and found there was a significantly higher C. I. in COPD BALF gradients compared to Control BALF gradients (Figure 3A). When comparing the neutrophil migration velocity, we did not observe any significant difference between groups (Figure 3B).

**Figure 4:**
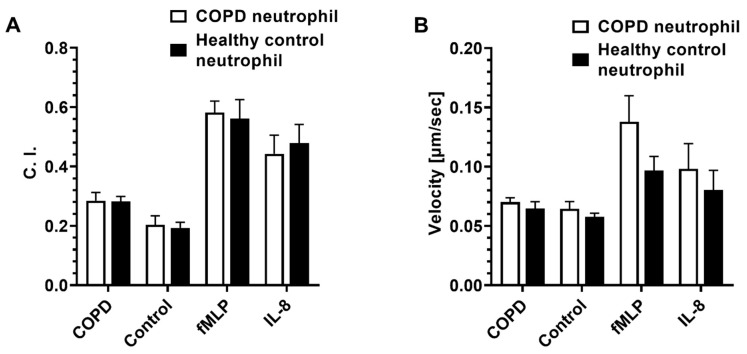
The migratory dynamics of peripheral neutrophils isolated from patients with COPD and healthy controls in different BALF samples and chemoattractants in the microfluidic device. Differential neutrophil migration C. I. (**A**) and velocity (**B**) in different BALF samples, fMLP, and IL-8 gradient fields. Data presented as mean ± SEM.

Next, we used neutrophils isolated from the blood of both COPD patients and healthy controls and compared their migratory dynamics in different BALF samples, fMLP, and IL-8 gradients. The results showed no significant difference in both C. I. and migration velocity between neutrophils from COPD patients and healthy controls for all conditions (Figure 4).

### 3.2. Effect of Dexamethasone on Neutrophil Chemotaxis

**Figure 5:**
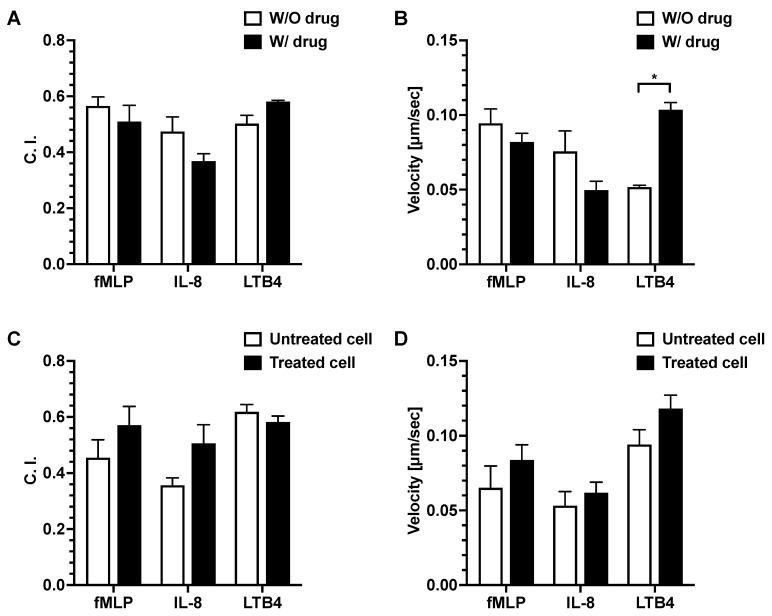
Effects of dexamethasone on neutrophil migration. Neutrophil migration C. I. (**A**) and velocity (**B**) of neutrophils in fMLP, IL-8, and LTB4 gradients with or without dexamethasone addition. Neutrophil migration C. I. (**C**) and velocity (**D**) in fMLP, IL-8, and LTB4 gradients using dexamethasone-treated and untreated neutrophils. The *p*-value for the two-group comparison was analyzed by an unpaired Student’s two-tailed *t*-test. *p* ≤ 0.05 (*) was considered statistically significant. Data presented as mean ± SEM. W/O, without; W/, with.

For the first set of experiments, where dexamethasone was diluted with the chemoattractant, the result showed that there was no significant difference in the neutrophil migration C. I. between the drug-added chemoattractant group and the chemoattractant alone group (without drug addition) (Figure 5A). There was a significantly higher neutrophil migration velocity in the LTB4 + drug group compared to the LTB4 alone group. No significant difference was observed in the neutrophil migration velocity between the drug-added chemoattractant group and the chemoattractant alone group for fMLP and IL-8 (Figure 5B).

For the second set of experiments, we pre-treated the neutrophils with dexamethasone, then examined their migration in different chemoattractant gradient fields. There was no significant difference in neutrophil migration C. I. or velocity between the drug-treated and untreated neutrophils in all chemoattractant gradients (Figure 5C,D).

### 3.3. Linear Regression Analyses of Neutrophil migration in COPD BALF against Patients’ Clinical Data

**Figure 6:**
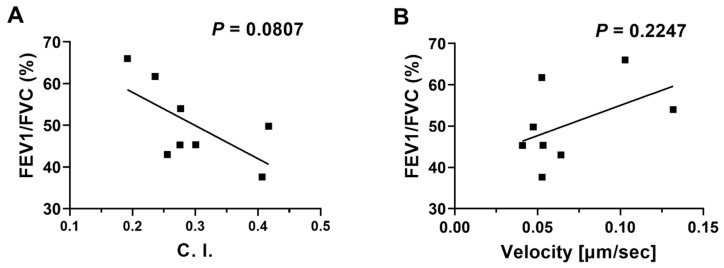
Linear regressions of neutrophil migration in COPD BALF samples against patients’ spirometry data. Linear regression of neutrophil migration C. I. (**A**) and velocity (**B**) in COPD BALF samples with FEV1/FVC (%). Data are presented as individual data points.

We compared the migratory dynamic data of neutrophil chemotaxis in COPD BALF with conventional COPD diagnosis parameters (i.e., the spirometry data). Although there was no significant relationship between the two sets of data, we noticed a trend of negative correlation between neutrophil migration C. I. in COPD BALF and FEV1/FVC (Figure 6A). Such a trend was not observed with neutrophil migration velocity (Figure 6B). We also compared the migratory dynamic data of neutrophil chemotaxis in COPD BALF with other clinical COPD diagnosis parameters (Supplementary Table S2). We found a negative correlation between neutrophil migration velocity and the COPD Assessment Test (CAT) score; however, such a relationship was not observed with neutrophil migration C. I. (Supplementary Table S2). Other than that, the migratory dynamic data of neutrophil chemotaxis in COPD BALF was not correlated with any clinical diagnosis parameters (Supplementary Table S2).

## 4. Discussion

In this study, we utilized a high-throughput microfluidic platform to investigate neutrophil chemotaxis in BALF samples and evaluated the feasibility of using this technique as a characterization tool for COPD. The significantly higher C. I. level generated in response to the COPD BALF gradient indicated that neutrophils had stronger chemotaxis in the COPD BALF compared to the Control BALF, suggesting that COPD BALF might have some chemoattractants that attract neutrophils to the lung from circulation. This result was consistent with the finding from a previous study, where it was found that sputum samples from COPD patients induce neutrophil chemotaxis on a microfluidic device [27]. Compared to sputum samples, BALF was collected directly from the lung, so it is more representative of the microenvironment within the lung tissue. The neutrophil migration C. I. values were lower in BALF samples compared to positive controls, including fMLP, IL-8, and LTB4, which were known as strong chemoattractants for neutrophils. This might be due to the relatively diluted concentration of chemoattractants in the BALF samples. Moreover, BALF samples might be composed of a multitude of chemoattractants that function differentially. IL-8 is secreted by macrophages and epithelial cells; its level was elevated in COPD BALF, and it is a major inflammatory factor in the BALF of COPD patients [43,44]. LTB4 is also involved in airway inflammation in COPD; it is released by macrophages and is another major neutrophil chemotactic agent in the airways of COPD patients [45]. Studies have shown that LTB4 concentrations are elevated in the BALF of COPD rats and in the sputum of COPD patients, suggesting that it may work synergistically with IL-8 to promote neutrophil chemotaxis [46,47,48]. Thus, they might be the main chemoattractants in COPD patients’ BALF that attract neutrophils; however, the exact chemoattractants involved in the pathophysiological mechanisms of COPD need to be further investigated.

There was no significant difference in the migratory dynamics of neutrophils isolated from the blood of patients with COPD and healthy controls in different BALF samples and chemoattractants. This result was in conflict with the previous study, where they studied neutrophil migration using the Dunn chamber and concluded peripheral neutrophils from COPD patients are intrinsically different than neutrophils isolated from healthy controls in terms of chemotactic behavior and migratory structure, with no difference in the surface expression of chemoattractant receptors [21]. The discrepancy between the findings of these two studies may be explained by the different chemotaxis platforms used. The Dunn chamber used in the previous study was not capable of establishing and maintaining a stable chemoattractant gradient, and neutrophils were added onto the coverslip, where they started to migrate from random spots. On the other hand, the microfluidic device used in this study has been well-validated for the feasibility of use in the chemotaxis experiments [37]. It contained a cell docking structure to align the cells at the low concentration area prior to the chemotactic migration and was capable of generating and maintaining an accurate and stable gradient in the migration channel using passive pumping and pressure-balancing strategies. Thus, the neutrophil migration trajectories were visualized more clearly for interpretation, and the chemotactic behaviors were more reliable in a stable gradient than using the Dunn chamber.

The trend of a negative relationship between neutrophil migration C. I. in COPD BALF and spirometry data suggested a potential correlation between the neutrophil migration and the severity of COPD, which was an expected result. The pathogenesis of COPD is mainly related to the overexpression of inflammatory mediators and cytokines and the activation of inflammatory signaling pathways that amplify the inflammatory response by recruiting inflammatory cells from the circulation to the lung. Thus, as the severity of COPD increases, there will be an elevated inflammatory response that results in neutrophil chemotaxis toward the lung tissue. This result was consistent with the findings from a previous study, where BALF levels of neutrophil-derived microvesicles are correlated with various key functional and clinically relevant COPD severity indexes [49]. As more neutrophils traveled to the lungs from the circulation, neutrophil levels in the BALF increased, which then led to airway inflammation and declining lung function. We also compared the chemotaxis results with other clinical diagnosis parameters and found that neutrophil chemotaxis velocity data was negatively correlated with CAT scores, but no other relationships were observed. CAT is a self-evaluation questionnaire on the impact of COPD on health status, with higher scores denoting a more severe impact on a patient’s life [50]. However, this result needs further validation due to the potentially subjective nature of the assessment of CAT scores and the relatively small number of subjects. We hope to recruit more subjects in the future to better understand the correlation between neutrophil chemotactic behaviors and clinical markers of COPD severity.

Dexamethasone is a synthetic glucocorticoid that is routinely used as a key intervention for COPD exacerbations by controlling airway inflammation [51,52,53]. We chose to perform two sets of dexamethasone drug response experiments as they represented different therapeutic approaches, both of which are currently being used to treat COPD patients. The direct dilution of dexamethasone with the chemoattractants IL-8, fMLP, and LTB4 simulated the inhalation drug treatment, whereas the dexamethasone-treated drug experiments simulated injections of the drug into patients, with the drug acting directly on cells. The only difference we found was that the neutrophil migration velocity was significantly higher in the LTB4 + drug group compared to the LTB4 alone group. Other than that, the result showed there was no significant difference in neutrophil migration C. I. and velocity between the treatment group and the control group for both sets of experiments. This suggested that neither the addition of dexamethasone to the chemoattractant nor the direct cell treatment had a significant effect on the migratory behaviors of neutrophils. The COPD patients recruited for this study had long-term clinically stable COPD, and therefore most of them had taken glucocorticoid medication. The result of this experiment demonstrated that increased neutrophil migration C. I. in COPD patients’ BALF was not related to the glucocorticoids medication. The neutrophils were pre-treated for 1 h in this experiment because we wanted to use them quickly after they were isolated from the blood to maintain their motility. In the future, we could pre-treat neutrophils for different time periods and see if there are any differences in their migratory behaviors.

We acknowledge that there are several limitations to our study. The first tube of BALF sample collected following PBS infusion was not used in this study because it was highly concentrated with mucus, which affected the establishment of the gradient field. Instead, we utilized relatively diluted BALF samples, and as a result, the level of chemoattractant might have been lower than it was supposed to be and might have influenced the result of neutrophil chemotaxis. To address this issue, we can collect the first draw of BALF samples and filter them through a microporous membrane after centrifugation to completely remove the mucus in the future. In addition, due to the difficulties in recruiting COPD patients, the subject size in this study was not very large, and the gender of COPD subjects was all male. We hope to recruit more COPD patients in the future to expand the subject size and be able to compare the differences in neutrophil migration in COPD BALF with different basic and clinical profiles. Although the result suggested increased neutrophil chemotaxis in COPD BALF, we were currently unable to identify the exact chemoattractant that causes neutrophil migration, and it would be of particular interest in the future to send COPD and Control BALF samples to specialized laboratories to identify different chemoattractant profiles.

## 5. Conclusions

In conclusion, this study demonstrated the feasibility of using a microfluidic platform to assess neutrophil chemotaxis in COPD BALF samples, and the directional neutrophil migration in COPD BALF was higher than that in Control BALF. This study also showed that the migratory behavior of neutrophils in three known chemoattractants (fMLP, IL-8, and LTB4) was not affected by glucocorticoid treatment, suggesting that this platform has the potential to be used to test drug responses and aid in the development of the targeted drug. In addition, the microfluidic device provided a useful technique that was capable of real-time observation of chemotactic migration, enabling accurate subsequent cell migration analysis, and could also be used for examining the migration of other cell types, such as lung cancer tumor cells and inflammatory cells involved in other airway inflammatory diseases (e.g., asthma). This fast, easy-to-use microfluidic platform may become a valuable tool for examining cellular function in COPD pathogenesis and assessing disease development.

## Figures and Tables

**Table 1:** The basic information and clinical data of the participants.

	No.	Gender	Age	FEV1 (L)	FEV1 (%)	FVC (L)	FVC (%)	FEV1/FVC (%)
COPD	1	M	61	0.82	29.60	2.18	60.30	37.63
	2	M	72	1.53	52.41	3.37	87.59	45.35
	3	M	59	1.76	54.92	2.85	70.45	61.74
	4	M	71	0.89	35.11	2.08	60.87	43.01
	5	M	68	0.79	40.51	1.75	70.23	45.32
	6	M	65	1.38	45.80	2.78	71.70	49.80
	7	M	67	1.55	59.07	2.87	85.13	54.01
	8	M	63	1.98	69.00	2.99	79.19	66.00
Control	1	F	53	2.13	91.80	2.8	102.20	76.16
	2	M	46	3.37	97.78	4.69	110.52	71.88
	3	M	40	3.72	97.63	4.5	97.37	82.80
	4	M	46	3.74	116.49	4.46	114.40	83.84
	5	M	36	3.69	99.14	4.45	99.79	82.96
	6	F	57	2.48	111.48	2.88	105.65	86.14
	7	M	38	3.65	95.65	4.49	97.87	81.16

## Data Availability

The data presented in this study are available upon reasonable request from the corresponding author.

## Supplementary Materials

The following supporting information can be downloaded at: https://www.mdpi.com/article/10.3390/mi14091740/s1, Table S1. The clinical data of COPD participants. Table S2. Linear regression analyses of neutrophil migration in COPD BALF samples against patients’ clinical data.

## References

[1] World Health Organization (2022). Chronic Obstructive Pulmonary Disease (COPD). https://www.who.int/news-room/fact-sheets/detail/chronic-obstructive-pulmonary-disease-(copd).

[2] Hoenderdos K., Condliffe A. (2013). The neutrophil in chronic obstructive pulmonary disease. Am. J. Respir. Cell Mol. Biol..

[3] Angelis N., Porpodis K., Zarogoulidis P., Spyratos D., Kioumis I., Papaiwannou A., Pitsiou G., Tsakiridis K., Mpakas A., Arikas S. (2014). Airway inflammation in chronic obstructive pulmonary disease. J. Thorac. Dis..

[4] Fricker M., Deane A., Hansbro P.M. (2014). Animal models of chronic obstructive pulmonary disease. Expert Opin. Drug Discov..

[5] Wright J.L., Cosio M., Churg A. (2008). Animal models of chronic obstructive pulmonary disease. Am. J. Physiol. Lung Cell. Mol. Physiol..

[6] Sutherland E.R., Martin R.J. (2003). Airway inflammation in chronic obstructive pulmonary disease. J. Allergy Clin. Immunol..

[7] Lowell F.C., Franklin W., Michelson A.L., Schiller I.W. (1956). Chronic obstructive pulmonary emphysema: A disease of smokers. Ann. Intern. Med..

[8] Halbert R.J., Natoli J.L., Gano A., Badamgarav E., Buist A.S., Mannino D.M. (2006). Global burden of COPD: Systematic review and meta-analysis. Eur. Respir. J..

[9] Harber P., Tashkin D.P., Simmons M., Crawford L., Hnizdo E., Connett J., Lung Health Study G. (2007). Effect of occupational exposures on decline of lung function in early chronic obstructive pulmonary disease. Am. J. Respir. Crit. Care Med..

[10] Barnes P.J. (2016). Inflammatory mechanisms in patients with chronic obstructive pulmonary disease. J. Allergy Clin. Immunol..

[11] Barnes P.J. (2014). Cellular and molecular mechanisms of chronic obstructive pulmonary disease. Clin. Chest Med..

[12] Rosales C. (2020). Neutrophils at the crossroads of innate and adaptive immunity. J. Leukoc. Biol..

[13] Kolaczkowska E., Kubes P. (2013). Neutrophil recruitment and function in health and inflammation. Nat. Rev. Immunol..

[14] Afonso P.V., Janka-Junttila M., Lee Y.J., McCann C.P., Oliver C.M., Aamer K.A., Losert W., Cicerone M.T., Parent C.A. (2012). LTB4 is a signal-relay molecule during neutrophil chemotaxis. Dev. Cell..

[15] Oved J.H., Paris A.J., Gollomp K., Dai N., Rubey K., Wang P., Spruce L.A., Seeholzer S.H., Poncz M., Worthen G.S. (2021). Neutrophils promote clearance of nuclear debris following acid-induced lung injury. Blood.

[16] Ham J., Kim J., Ko Y.G., Kim H.Y. (2022). The Dynamic Contribution of Neutrophils in the Chronic Respiratory Diseases. Allergy Asthma Immunol. Res..

[17] Hogg J.C., Chu F., Utokaparch S. (2004). The nature of small-airway obstruction in chronic obstructive pulmonary disease. N. Engl. J. Med..

[18] Pesci A., Majori M., Cuomo A., Borciani N., Bertacco S., Cacciani G., Gabrielli M. (1998). Neutrophils infiltrating bronchial epithelium in chronic obstructive pulmonary disease. Respir. Med..

[19] Pilette C., Colinet B., Kiss R., Andre S., Kaltner H., Gabius H.J., Delos M., Vaerman J.P., Decramer M., Sibille Y. (2007). Increased galectin-3 expression and intra-epithelial neutrophils in small airways in severe COPD. Eur. Respir. J..

[20] Donaldson G.C., Seemungal T.A., Patel I.S., Bhowmik A., Wilkinson T.M., Hurst J.R., Maccallum P.K., Wedzicha J.A. (2006). Airway and systemic inflammation and decline in lung function in patients with COPD. Respir. Med. COPD Update.

[21] Sapey E., Stockley J.A., Greenwood H., Ahmad A., Bayley D., Lord J.M., Insall R.H., Stockley R.A. (2011). Behavioral and structural differences in migrating peripheral neutrophils from patients with chronic obstructive pulmonary disease. Am. J. Respir. Crit. Care Med..

[22] Stănescu D., Sanna A., Veriter C., Kostianev S., Calcagni P.G., Fabbri L.M., Maestrelli P. (1996). Airways obstruction, chronic expectoration, and rapid decline of FEV1 in smokers are associated with increased levels of sputum neutrophils. Thorax.

[23] Bartoli M.L., Di Franco A., Vagaggini B., Bacci E., Cianchetti S., Dente F.L., Tonelli M., Paggiaro P.L. (2009). Biological markers in induced sputum of patients with different phenotypes of chronic airway obstruction. Respiration.

[24] Lacoste J.Y., Bousquet J., Chanez P., Van Vyve T., Simony-Lafontaine J., Lequeu N., Vic P., Enander I., Godard P., Michel F.B. (1993). Eosinophilic and neutrophilic inflammation in asthma, chronic bronchitis, and chronic obstructive pulmonary disease. J. Allergy Clin. Immunol..

[25] Rytila P., Plataki M., Bucchieri F., Uddin M., Nong G., Kinnula V.L., Djukanovic R. (2006). Airway neutrophilia in COPD is not associated with increased neutrophil survival. Eur. Respir. J..

[26] Singh D., Edwards L., Tal-Singer R., Rennard S. (2010). Sputum neutrophils as a biomarker in COPD: Findings from the ECLIPSE study. Respir. Res..

[27] Wu J., Hillier C., Komenda P., Lobato de Faria R., Levin D., Zhang M., Lin F. (2015). A Microfluidic Platform for Evaluating Neutrophil Chemotaxis Induced by Sputum from COPD Patients. PLoS ONE.

[28] Lonergan M., Dicker A.J., Crichton M.L., Keir H.R., Van Dyke M.K., Mullerova H., Miller B.E., Tal-Singer R., Chalmers J.D. (2020). Blood neutrophil counts are associated with exacerbation frequency and mortality in COPD. Respir. Res..

[29] Franz C.M., Jones G.E., Ridley A.J. (2002). Cell Migration in Development and Disease. Dev. Cell.

[30] Luster A.D., Alon R., von Andrian U.H. (2005). Immune cell migration in inflammation: Present and future therapeutic targets. Nat. Immunol..

[31] Woolhouse I., Bayley D., Stockley R. (2002). Sputum chemotactic activity in chronic obstructive pulmonary disease: Effect of α1–antitrypsin deficiency and the role of leukotriene B4 and interleukin 8. Thorax.

[32] Beeh K.M., Kornmann O., Buhl R., Culpitt S.V., Giembycz M.A., Barnes P.J. (2003). Neutrophil chemotactic activity of sputum from patients with COPD: Role of interleukin 8 and leukotriene B4. Chest.

[33] Boyden S. (1962). The Chemotactic Effect of Mixtures of Antibody and Antigen on Polymorphonuclear Leucocytes. J. Exp. Med..

[34] Zicha D., Dunn G.A., Brown A.F. (1991). A new direct-viewing chemotaxis chamber. J. Cell Sci..

[35] Ren J., Wang N., Guo P., Fan Y., Lin F., Wu J. (2022). Recent advances in microfluidics-based cell migration research. Lab Chip.

[36] Wu J., Wu X., Lin F. (2013). Recent developments in microfluidics-based chemotaxis studies. Lab Chip.

[37] Wu J., Kumar-Kanojia A., Hombach-Klonisch S., Klonisch T., Lin F. (2018). A radial microfluidic platform for higher throughput chemotaxis studies with individual gradient control. Lab Chip.

[38] Campbell J.M., Balhoff J.B., Landwehr G.M., Rahman S.M., Vaithiyanathan M., Melvin A.T. (2018). Microfluidic and Paper-Based Devices for Disease Detection and Diagnostic Research. Int. J. Mol. Sci..

[39] Lin F., Butcher E.C. (2006). T cell chemotaxis in a simple microfluidic device. Lab Chip.

[40] Sackmann E.K., Berthier E., Schwantes E.A., Fichtinger P.S., Evans M.D., Dziadzio L.L., Huttenlocher A., Mathur S.K., Beebe D.J. (2014). Characterizing asthma from a drop of blood using neutrophil chemotaxis. Proc. Natl. Acad. Sci. USA.

[41] Stockley J.A., Walton G.M., Lord J.M., Sapey E. (2013). Aberrant neutrophil functions in stable chronic obstructive pulmonary disease: The neutrophil as an immunotherapeutic target. Int. Immunopharmacol..

[42] Liu Y., Ren X., Wu J., Wilkins J.A., Lin F. (2022). T Cells Chemotaxis Migration Studies with a Multi-Channel Microfluidic Device. Micromachines.

[43] Zhang X., Zheng H., Zhang H., Ma W., Wang F., Liu C., He S. (2011). Increased interleukin (IL)-8 and decreased IL-17 production in chronic obstructive pulmonary disease (COPD) provoked by cigarette smoke. Cytokine.

[44] Wang Y., Xu J., Meng Y., Adcock I.M., Yao X. (2018). Role of inflammatory cells in airway remodeling in COPD. Int. J. Chron. Obstruct. Pulm. Dis..

[45] Finney-Hayward T.K., Bahra P., Li S., Poll C.T., Nicholson A.G., Russell R.E., Ford P.A., Westwick J., Fenwick P.S., Barnes P.J. (2009). Leukotriene B4 release by human lung macrophages via receptor- not voltage-operated Ca^2+^ channels. Eur. Respir. J..

[46] Hill A.T., Bayley D., Stockley R.A. (1999). The interrelationship of sputum inflammatory markers in patients with chronic bronchitis. Am. J. Respir. Crit. Care Med..

[47] Barnes P. (2003). Chronic obstructive pulmonary disease • 12: New treatments for COPD. Thorax.

[48] Ding Y.L., Yao W.Z., Zheng J., Zhu Y.L., Liu Z. (2005). Changes of leukotriene B4 in chronic obstructive pulmonary disease and effects of theophylline on leukotriene B4. Beijing Da Xue Xue Bao Yi Xue Ban.

[49] Wang C., Zhou J., Wang J., Li S., Fukunaga A., Yodoi J., Tian H. (2020). Progress in the mechanism and targeted drug therapy for COPD. Signal Transduct. Target. Ther..

[50] Jones P.W., Harding G., Berry P., Wiklund I., Chen W.H., Kline Leidy N. (2009). Development and first validation of the COPD Assessment Test. Eur. Respir. J..

[51] Ardestani M.E., Kalantary E., Samaiy V., Taherian K. (2017). Methyl prednisolone vs Dexamethasone in Management of COPD Exacerbation; a Randomized Clinical Trial. Emergency.

[52] Moslem S., Hossein D., Afrasiabifar A., Ahad H. (2022). Evaluation of Effectiveness of Dexamethasone versus Hydrocortisone in Reducing Hospital Length, Peak Expiratory Flow, Oxidative Stress Factors and Inflammatory Mediators in Chronic Obstructive Pulmonary Disease (COPD) Exacerbation Patients: A Randomized Controlled Trial. Res. Sq..

[53] Grundy S., Plumb J., Kaur M., Ray D., Singh D. (2016). Additive anti-inflammatory effects of corticosteroids and phosphodiesterase-4 inhibitors in COPD CD8 cells. Respir. Res..

