# Supplementary material for: Bronchoalveolar Lavage Fluid from Chronic Obstructive Pulmonary Disease Patients Increases Neutrophil Chemotaxis Measured by a Microfluidic Platform
Source: Micromachines (Basel). 2023 Sep 6;14(9):1740. doi: 10.3390/mi14091740 (PMC10537285; doi:10.3390/mi14091740)
Supplement: Supplementary file 1 [file micromachines-14-01740-s001.zip › micromachines-2567634-supplementary.pdf]

Supporting Information

Bronchoalveolar Lavage Fluid from Chronic Obstructive Pulmonary Disease Patients Increases Neutrophil Chemotaxis Measured by a Microfluidic Platform

Jiaqi Ren [1,†], Wenfang Chen [2,†], Zhicheng Zhong [2,†], Ning Wang [1,3], Xi Chen [1], Hui Yang [1], Jing Li [2], Ping Tang [2], Yanping Fan [3], Francis Lin [4], Changqing Bai [2,*] and Jiandong Wu [1,*]

[1] Institute of Biomedical and Health Engineering, Shenzhen Institute of Advanced Technology, Chinese Academy of Sciences, Shenzhen 518055, China
[2] Department of Pulmonary and Critical Care Medicine, Shenzhen University General Hospital, Shenzhen 518055, China
[3] School of Optical-Electrical and Computer Engineering, University of Shanghai for Science and Technology, Shanghai 200093, China
[4] Department of Physics and Astronomy, University of Manitoba, Winnipeg, MB R3T 2N2, Canada
* Correspondence: baicq307@163.com (C.B.); jd.wu@siat.ac.cn (J.W.)
† These authors contributed equally to this work.

Supplementary Table S1. The clinical data of COPD participants.

Patient No.	CAT score	Frequency of COPD (times/year)	Years of onset	mMRC Dyspnea Scale	GOLD stage	pO_2 (mmHg)	pCO_2 (mmHg)	pH	WBC (10^9 L^{-1})	Neutrophil (10^9 L^{-1})
1	20	1	10	1	3	69	45.9	7.38	6.96	4.47
2	15	1	3	1	2	51	38.6	7.423	7.15	4.31
3	21	1	10	1	2	69.7	33.3	7.4	9.2	6.5
4	15	1	22	1	3	82	42	7.429	10.2	8
5	22	2	30	3	3	79	44.3	7.428	4.83	2.45
6	11	1	2	1	2	64	32.4	7.473	4.71	3.41
7	8	1	30	0	2	81	34.5	7.37	7.21	4.59
8	11	2	20	2	2	68	41.6	7.422	5.62	3.52

Supplementary Table S2. Linear regression analyses of neutrophil migration in COPD BALF samples against patients' clinical data. $p \leq 0.05$ (*) was considered statistically significant. mMRC, Modified Medical Research Council; GOLD, Global Initiative for Chronic Obstructive Lung Disease; CAT, COPD Assessment Test; WBC, white blood cell.

**Variables**	**Equation**	**r^2**	***p***
Velocity vs. COPD patients' clinical data			
Velocity vs. FEV%	Y = 257.8 × X + 30.74	0.3976	0.0937
Velocity vs. FVC%	Y = 161.5 × X + 62.18	0.2586	0.1982
Velocity vs. CAT score	Y = −120.6 × X + 23.59	0.5484	0.0356*
Velocity vs. frequency of COPD	Y = 1.029 × X + 1.180	0.005055	0.8671
Velocity vs. years of onset	Y = 181.0 × X + 3.539	0.2663	0.1905
Velocity vs. mMRC Dyspnea Scale	Y = −11.68 × X + 2.046	0.1775	0.2986
Velocity vs. GOLD Stage	Y = −6.541 × X + 2.821	0.1633	0.3208
Velocity vs. pH	Y = −0.5011 × X + 7.450	0.2457	0.2116
Velocity vs. pO_2 (mmHg)	Y = 106.9 × X + 63.18	0.1096	0.4231
Velocity vs. pCO_2 (mmHg)	Y = −31.65 × X + 41.23	0.03815	0.6430
Velocity vs. WBC (10^9 L^{-1})	Y = 3.250 × X + 6.764	0.002800	0.9010
Velocity vs. neutrophil (10^9 L^{-1})	Y = 1.126 × X + 4.579	0.0004044	0.9623
C. I. vs. COPD patients' clinical data			
C. I. vs. FEV%	Y = −100.9 × X + 78.08	0.3729	0.1078
C. I. vs. FVC%	Y = −38.10 × X + 84.43	0.08800	0.4756
C. I. vs. CAT score	Y = 2.767 × X + 14.56	0.001765	0.9213
C. I. vs. frequency of COPD	Y = −2.802 × X + 2.077	0.2291	0.2302
C. I. vs. years of onset	Y = −74.90 × X + 37.97	0.2788	0.1786
C. I. vs. mMRC Dyspnea Scale	Y = −2.827 × X + 2.084	0.06360	0.5468
C. I. vs. GOLD Stage	Y = 1.201 × X + 2.021	0.03370	0.6634
C. I. vs. pH	Y = 0.07768 × X + 7.393	0.03611	0.6522
C. I. vs. pO_2 (mmHg)	Y = −36.13 × X + 81.12	0.07657	0.5070
C. I. vs. pCO_2 (mmHg)	Y = −2.502 × X + 39.81	0.001458	0.9285
C. I. vs. WBC (10^9 L^{-1})	Y = −8.189 × X + 9.401	0.1087	0.4251
C. I. vs. neutrophil (10^9 L^{-1})	Y = −5.812 × X + 6.371	0.06586	0.5395
